# A lightweight magnetically shielded room with active shielding

**DOI:** 10.1038/s41598-022-17346-1

**Published:** 2022-08-09

**Authors:** Niall Holmes, Molly Rea, James Chalmers, James Leggett, Lucy J. Edwards, Paul Nell, Stephen Pink, Prashant Patel, Jack Wood, Nick Murby, David Woolger, Eliot Dawson, Christopher Mariani, Tim M. Tierney, Stephanie Mellor, George C. O’Neill, Elena Boto, Ryan M. Hill, Vishal Shah, James Osborne, Rosemarie Pardington, Peter Fierlinger, Gareth R. Barnes, Paul Glover, Matthew J. Brookes, Richard Bowtell

**Affiliations:** 1 Sir Peter Mansfield Imaging Centre, School of Physics and Astronomy, University of Nottingham, University Park, Nottingham, NG7 2RD UK; 2 Magnetic Shields Limited, Headcorn Road, Staplehurst, Tonbridge, Kent, TN12 0DS UK; 3 Cerca Magnetics Limited, Headcorn Road, Staplehurst, Tonbridge, Kent, TN12 0DS UK; 4 Wellcome Centre for Human Neuroimaging, UCL Institute of Neurology, London, WC1N 3AR UK; 5 QuSpin Inc., 331 South 104th Street, Suite 130, Louisville, CO 80027 USA; 6 Young Epilepsy, St. Piers Lane, Lingfield, Surrey, RH7 6PW UK; 7 Department of Physics, Technical University Munich, 85748 Garching, Germany

**Keywords:** Applied physics, Medical imaging, Brain imaging, Neuroscience

## Abstract

Magnetically shielded rooms (MSRs) use multiple layers of materials such as MuMetal to screen external magnetic fields that would otherwise interfere with high precision magnetic field measurements such as magnetoencephalography (MEG). Optically pumped magnetometers (OPMs) have enabled the development of wearable MEG systems which have the potential to provide a motion tolerant functional brain imaging system with high spatiotemporal resolution. Despite significant promise, OPMs impose stringent magnetic shielding requirements, operating around a zero magnetic field resonance within a dynamic range of ± 5 nT. MSRs developed for OPM-MEG must therefore effectively shield external sources and provide a low remnant magnetic field inside the enclosure. Existing MSRs optimised for OPM-MEG are expensive, heavy, and difficult to site. Electromagnetic coils are used to further cancel the remnant field inside the MSR enabling participant movements during OPM-MEG, but present coil systems are challenging to engineer and occupy space in the MSR limiting participant movements and negatively impacting patient experience. Here we present a lightweight MSR design (30% reduction in weight and 40–60% reduction in external dimensions compared to a standard OPM-optimised MSR) which takes significant steps towards addressing these barriers. We also designed a ‘window coil’ active shielding system, featuring a series of simple rectangular coils placed directly onto the walls of the MSR. By mapping the remnant magnetic field inside the MSR, and the magnetic field produced by the coils, we can identify optimal coil currents and cancel the remnant magnetic field over the central cubic metre to just |**B**|= 670 ± 160 pT. These advances reduce the cost, installation time and siting restrictions of MSRs which will be essential for the widespread deployment of OPM-MEG.

## Introduction

Low magnetic field environments, such as magnetically shielded rooms (MSRs), with minimal disturbances from external sources are needed for precision experiments, including the search for the electric dipole moment of fundamental particles^1^, and biomagnetic recordings, such as magnetoencephalography (MEG)^2^. MEG is a non-invasive functional brain imaging technique which measures magnetic fields generated by neuronal currents^3^. Inverse modelling is applied to these measured fields to reconstruct the underlying neuronal activity with excellent spatial (~ 3 mm) and temporal (~ 1 ms) resolution^2,4^, offering a unique and non-invasive window into the function of the human brain^5^. However, the neuromagnetic field is on the order of 100 s of femtotesla (fT) at the scalp and so is easily masked by interfering sources. A MSR is therefore a critical component of a MEG system^2^.

State-of-the-art MEG scanners use a fixed array of superconducting quantum interference devices (SQUIDs). As these sensors must be cooled to liquid helium temperatures, the geometry of a SQUID-MEG MSR is largely governed by the requirement that a cryogenic dewar be sited within the shield. However, recent developments in quantum technologies have led to MEG systems based on optically pumped magnetometers (OPMs)^6–8^. Commercially available OPMs (such as those provided by QuSpin Inc. (Louisville, Colorado, USA) and FieldLine Inc. (Boulder, Colorado, USA)) are small, integrated, magnetic field sensors which exploit the quantum properties of alkali metals^9,10^. These sensors can be mounted into a wearable helmet which allows participants to move during MEG studies^11^. To attain the level of sensitivity required to measure MEG signals (signals of interest in the 1–100 Hz range, sensitivity of < 15 fT/√Hz required) OPMs are operated around a zero magnetic field resonance, within a narrow dynamic range of ± 5 nT^12^ and a bandwidth of 0–130 Hz. MSRs for OPM-MEG must therefore screen magnetic interference from sources within this frequency range, whilst also providing an environment in which magnetic fields are < 1 nT in magnitude and the gradients in the field are < 1 nT/m. This performance is required over a volume large enough to contain both the head and the sensor array during the expected range of participant movements, such that any change in field (either induced by an external source, or via rotation/translation of a sensor during participant movement) does not send any OPM outside its dynamic range.

In contrast to SQUID-MEG, wearable OPM-MEG can be carried out using a wide range of MSR shapes and sizes. As well as fixed cuboidal shields, cylindrical shields that can be easily relocated have been used where space is limited (though such designs largely prohibit participant motion)^7^. To realise the full potential of OPM-MEG and enable widespread deployment, MSRs must be optimised to provide the magnetic environment required for OPM operation whilst addressing key concerns such as the cost, weight, comfort and architectural impact of the shield.

Passive shielding of magnetic fields is achieved by enclosing experiments within multiple layers of a material with a high magnetic permeability. A commonly used material is MuMetal, which is a nickel–iron alloy of very high permeability ($\mu_{r}$ can be greater than 200,000 following a heat treatment to enlarge material grain). The flux-shunting mechanism shields low frequency (DC to 10 Hz) magnetic fields by diverting flux lines into the MuMetal where they follow the MuMetal around the shielded region and exit on the other side of the enclosure^13^. To screen high frequency (10 Hz to MHz) magnetic fields, a material with a high electrical conductivity is also used (e.g. copper or aluminium). Eddy-currents in the material induce a magnetic field which deflects the imposed field^13^. Current commercially available shielded rooms which are optimised for OPM-MEG employ four layers of MuMetal, and one layer of copper. As a result, these MSRs are heavy (> 10 tonnes), with strict siting requirements and a need for substantial building work. This coupled with long manufacturing and installation times makes further innovation in MSR design highly desirable.

Although a high shielding factor (the ratio of the magnetic field strength of the interfering source measured with and without the shield) can be achieved when screening external sources, the ferromagnetic nature of MuMetal means MSRs often have a remnant, internal magnetic field of around 10–30 nT^14^. Demagnetisation coils can be incorporated into the MuMetal walls of the MSR to reduce the remnant field so, if a decaying sinusoidal current is applied to these coils, the metal is driven around its $B {-} H$ curve towards a point of zero magnetisation^15,16^. The remnant magnetic field is then reduced to a level which depends on material choices (e.g. layer thickness, layer spacing and material permeability), and engineering imperfections (such as access holes, doors and joints between MuMetal panels). A field strength of around 2–5 nT is typically achieved^17^.

To compensate the remnant field further (and enable participant movement in OPM-MEG) active magnetic shielding is employed where electromagnetic coils are used to generate a magnetic field which is equal in magnitude, but opposite in direction, to the remnant field. Tri-axial Helmholtz coils, or similar systems, can be used to generate known magnetic fields in all orientations and compensate for remnant field and field gradients^8^, but these designs are ill-suited for use with human subjects as they enclose participants in an uncomfortable setting. Our previous work demonstrated the first motion-tolerant OPM-MEG studies, using bi-planar ‘fingerprint’ coils. These coils are produced using MRI gradient coil design methods which restricted the coil windings to two large planes (1.6 × 1.6 m^2^ square planes separated by 1.5 m). These can be placed on either side of a participant, allowing easy access^18^. Such systems occupy space inside the MSR, and field cancellation is limited to a small (0.4 × 0.4 × 0.4 m^3^) volume at the centre of the space between the coil planes. Furthermore, although the coils in these systems are typically designed to generate a distinct homogeneous uniform field or field gradient over a desired volume, the produced fields interact with the MuMetal walls of the MSR leading to a change in the expected field strength per unit current and a distortion of the spatial variation of the magnetic field, making accurate cancellation challenging if interactions are not accounted for at the design stage^19–21^. It is of course desirable to move coil wirepaths closer to the walls of the MSR to maximise the available space inside, though this increases the strength of the interaction with the MuMetal.

Here we describe the design, construction, operation and performance of a passively and actively magnetically shielded room—with internal dimensions 2.4 × 2.4 × 2.4 m^3^—which makes significant steps towards overcoming the challenges outlined above. The light MSR is formed from two layers of MuMetal and one layer of copper, with reduced layer spacings compared to previous designs. Demagnetisation coils are incorporated into the MuMetal layers. To improve the shielding efficiency of the lighter MSR, we developed a new multi-coil active shielding system, the ‘window coil’, featuring a series of 27 rectangular coils. The coil dimensions and positions were optimised, accounting for interaction with MuMetal walls of the MSR, and the constructed coils placed directly onto the MSR’s walls to maximise the available space in the MSR, and significantly improve the experience of participants in MEG experiments. By mapping the strength and spatial variation of the magnetic field produced by each coil, and also mapping the remnant magnetic field, appropriate coil currents were identified to generate a superposition of magnetic field patterns from all 27 coils that cancels the remnant field over a large (1 × 1 × 1 m^3^) volume inside the MSR. The shielding performance of the MSR was validated by measuring shielding factors over a range of frequencies using controlled magnetic fields. Example OPM sensor noise data in the empty MSR were also collected to verify its suitability for biomagnetic recordings.

## Methods

### Magnetically shielded room

The MSR described here was installed at Young Epilepsy (Lingfield, Surrey, UK) a charity for children and young adults with epilepsy. The MSR was designed for use by an OPM-MEG system (Cerca Magnetics Limited, Kent, UK) in epilepsy research. As well as ensuring magnetic performance, aesthetic considerations were important to create a comfortable scanning environment for young patients and their families.

#### MSR design

Magnetic Shields Limited (MSL, Kent, UK) designed and constructed the lightweight MSR (The Light MuRoom) which comprises two layers (outer and inner layers) of 1.5 mm thick MuMetal, and one layer (middle layer) of 4 mm-thick copper. This is a significant reduction in the amount of shielding material compared to existing OPM-optimised MSRs such as the MSL standard MuRoom^22^ at the Sir Peter Mansfield Imaging Centre, University of Nottingham (UoN) which features two outer layers of 1.5 mm thick MuMetal, two inner layers of 1 mm thick MuMetal and one middle layer of 6 mm copper. The modifications to the standard MuRoom design result in a 40% reduction in MuMetal and 33% reduction in copper reduces the total weight of the MSR by around 30% to ~ 7 tonnes. The material cost and the required installation time are also decreased.

To allow for construction within laboratory and non-laboratory environments where space is constrained, the total distance between the inner and outer layers was also reduced. The standard MuRoom has an internal volume (x,y,z) (y is the vertical direction) of 3 × 2.4 × 3 m^3^, and an external volume of 3.7 × 3.4 × 4.0 m^3^ whereas the Light MuRoom has a usable internal volume of 2.4 × 2.4 × 2.4 m^3^ and an external volume of approximately 2.8 × 3 × 2.8 m^3^; a reduction of between 40 and 60% in the total wall thickness along the different dimensions of the MSR. The spacing between the outer MuMetal and copper layer is ~ 0.1 m, and the spacing between the copper and inner MuMetal layer is ~ 0.02 m.

The MSR was constructed from MuMetal and copper panels mounted onto an aluminium framework which was placed on an anti-vibrational layer on a poured concrete base. The largest MuMetal panels were 1.11 × 0.55 m^2^ and the largest copper panels were 0.76 × 0.76 m^2^. This was an approximately 20% reduction in area compared to the 1.23 × 0.61 m^2^ MuMetal panels and a 40% reduction compared to the 1 × 1 m^2^ copper panels used in the UoN MuRoom. A reduction in MuMetal panel size improves production efficiency, as more panels can undergo heat treatment in a single run, reducing fabrication time and costs. Smaller panels are also easier to handle during installation. The MuMetal shielding panels in the outer layer were arranged at 90° in plane rotation to those on the inner layer to minimise flux leakage. The MSR door comprises three hinges and features a lever-style handle. An emergency release mechanism allows access to the MSR in under twenty seconds in the unlikely event of a failure of the standard locking mechanism, critical to obtain patient access in the event of a seizure. LED lighting was placed along the edges of the MSR to ensure minimal DC field shifts at the centre of the room.

**Figure 1:**
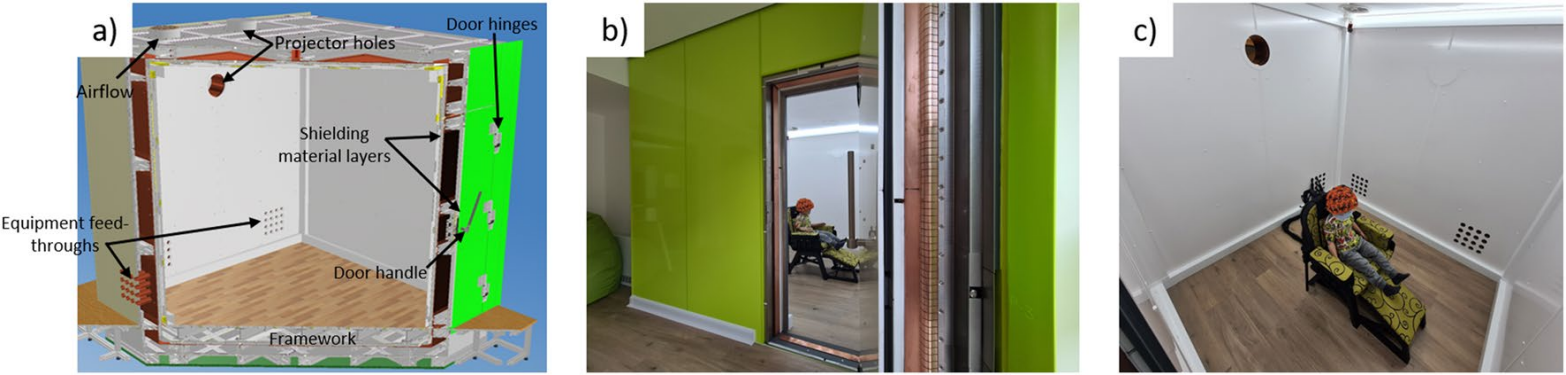
The Light MuRoom magnetically shielded room. (**a**) Computer model of the Light MuRoom, cross-section revealing framework and layer structure. (**b**) Photograph of the exterior of the participant facing side of the MSR. (**c**) Photograph of the interior of the MSR with wearable OPM-MEG system mounted on a plastic mannequin.

Consultation sessions were held with the health professionals and patient groups who would use the MSR to determine how to provide a comfortable scanning environment. The MSR was sunk into the floor, so that participants do not have to climb a step upon entry. Building works were carried out post-installation so that the front side of the MSR is a continuation of the room in which it is situated, rather than a separate entity. To the participant, only the front wall of the MSR is visible; a separate room behind the wall contains the rear of the MSR and is used for housing equipment. Furthermore, the flooring in the waiting lobby was the same floor as in the MSR. These elements create a calm environment for a recording. An annotated computer-generated model and photographs of the constructed MSR are shown in Fig. 1.

#### Demagnetisation

Magnetisation of MuMetal increases over time due to external field fluctuations such as those manifested by the Earth’s magnetic field. This effect is exacerbated by opening and closing the door of the MSR due to the magnetic domains within the material moving through, and aligning with, the Earth’s field. To generate a repeatable MuMetal magnetisation, demagnetisation coils were wound around each face of the inner and outer layers of MuMetal, with windings balanced on either side of the layers^16^. The coils of the two layers were connected in series with each other. Currents were applied to the coils via a digital to analogue converter (DAC) on a National Instruments (NI, Austin, Texas, USA) BNC-6212 digital acquisition (DAQ) unit which outputs to an AE Techron (Elkhart, Indiana, USA) 7226 power amplifier that was connected to one side of a Bel (Lynbrook, New York, USA) 530-SU-7.5 signal transformer (to remove any DC offset). The other side of the transformer was connected to the demagnetisation coils. A linearly decaying sinusoidal signal is generated by interfacing the DAQ with a LabVIEW (NI) program. The optimal waveform parameters (frequency = 9.5 Hz, peak current ~ 1.5 A, decaying for 60 s) were found empirically by measuring the magnetic field before and after demagnetisation with a Bartington (Mag-13, Bartington Instruments, Witney, UK) triaxial fluxgate magnetometer. Demagnetisation is performed every time the door is opened, and the amplifier circuit is switched off prior to any measurements to avoid interference.

The vertical component of the remnant magnetic field magnitude post demagnetisation (measured using the fluxgate magnetometer at the centre of the MSR and at the corners of a 0.4 × 0.4 × 0.4 m^3^ volume, prior to any active magnetic shielding) was 4.84 ± 0.39 nT (mean and standard deviation, max/min value of 4.34/5.70 nT, shielding of Earth’s magnetic field by a factor of ~ 10,000). For comparison, the standard MuRoom achieves a remnant field < 2 nT at UoN^22^. Additional active magnetic shielding was developed to further compensate the remnant field (see “Results” section).

#### Shielding factor measurements

To quantify the performance of the MSR, shielding factor measurements were taken by applying known magnetic fields over a range of frequencies pre- and post-installation, using the fluxgate magnetometer interfaced with a Bartington Spectramag-6 24-bit DAQ to record data. Pre-installation, a square, 5-turn, electromagnetic coil (side length 3.3 m offset, 500 mm from the planned location of the exterior walls, produced field oriented vertically from floor to ceiling) was set up around the perimeter of the MuRoom foundations in the floor. The coil was driven with sinusoidal waveforms at frequencies of 0.01, 0.1, 1, 10, and 100 Hz using an AE Techron 7226 power amplifier. Waveforms were generated using custom Python software and outputted by a NI USB-6212 DAQ. A coil current of approximately 2 A pk--pk generated a sinusoidal magnetic field with an amplitude of 20 µT pk--pk at the centre of the coil at each frequency. Field amplitudes were measured at a point corresponding to the internal centre-point of the MuRoom post-construction. Once the MSR installation was complete, the coil was rebuilt, and the magnetic field measurements were repeated. Amplitudes of the AC signals were taken from a fast Fourier transform of the recorded data. The ratios of the field values measured with and without the MSR were used to estimate the shielding factor of the MSR.

### Window coil active magnetic shielding system

To reinforce the effects of the passive shielding and demagnetisation coils, we developed an active magnetic shielding system which we refer to as the ‘window coil’. The window coil set was parameterised, and an approach adapted from MRI multi-coil shimming systems^23,24^ was used to optimise coil parameters within the constraints set by the MSR geometry and the driving electronics^25^.

#### Coil design

**Figure 2:**
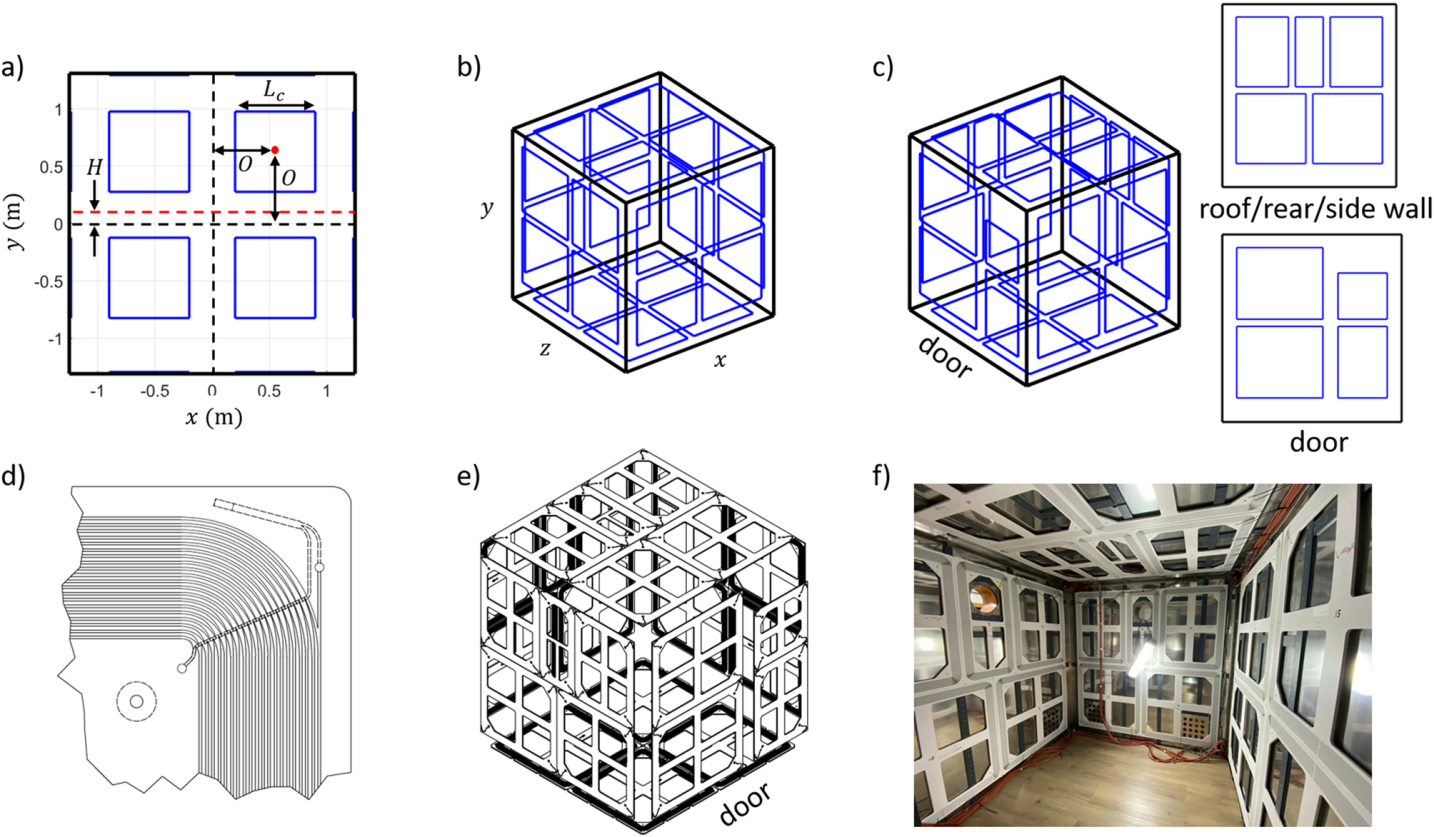
The window coil active magnetic shielding system. (**a**) Parameterisation of the window coil. Four square coils are arranged with four-fold symmetry on each face. The coil square side length, offset from the centre of the window and offset in the vertical axis are shared between all faces. Coil parameters are optimised to produce known components of magnetic field over the central cubic metre of the MSR. (**b**) Final optimised window coil featuring 24 identical square coils. This structure is challenging to engineer due to the need to incorporate the access door and projection ports in 3/6 walls. (**c**) Adapted design accounts for geometry of the MSR and features 27 coils. (**d**) Drawing of the corner of a single coil panel. To accommodate 20 turns of wire in each coil, a series of grooves are arranged in a spiral pattern into which copper wire is placed. The conductor return path is also shown. (**e**) Final model of the window coil featuring all panels. (**f**) Photograph of installed coil panels (floor coils hidden beneath the flooring and door coils hidden by angle of photograph) taken prior to cladding.

The window coil system comprises six sets of four, square, electromagnetic unit-coils, each formed of twenty turns of wire, arranged with four-fold symmetry on a single face of the MSR. The coil arrangement on each MSR face is characterised by three parameters:

- $L_{c}$, the square-side-length of each unit coil in the window coil,
- $O$, the offset of the centre of each square unit coil from the centre of the window coils,
- $H$, the offset of the centre of the window coil from the centre of the MuMetal wall in the vertical ($y$-direction). The coil sets are centred in the horizontal ($x$ and $z$) dimensions of each wall, as outlined in Fig. 2a. For ease of manufacturing, the same values of the $O$ and $L_{c}$ parameters were used for all walls of the MSR. To inform the choice of an optimal set of coil parameters, we conducted a simulation study to investigate the ability of a range of different coil designs to produce a desired magnetic field over a series of ‘target points’ within the MSR.

The magnetic field at a target point ${\varvec{r}}_{{\varvec{n}}} \left( {x,y,z} \right)$ produced by a square unit coil $m$ (from a set of $M = 24$ unit coils), when carrying a unit current, is denoted by ${\varvec{b}}_{{\varvec{m}}} \left( {{\varvec{r}}_{{\varvec{n}}} } \right)$. The total magnetic field, ${\varvec{B}}\left( {{\varvec{r}}_{{\varvec{n}}} } \right)$ at this target point from all coils in the system, with the $m^{th}$ coil carrying a current $i_{m}$ (from $m = 1$ up to coil $m = M$), is the vector sum of all coil contributions i.e.

1 $${\varvec{B}}\left( {{\varvec{r}}_{{\varvec{n}}} } \right) = \mathop \sum \limits_{m = 1}^{M} i_{m} {\varvec{b}}_{{\varvec{m}}} \left( {{\varvec{r}}_{{\varvec{n}}} } \right).$$

Expanding the calculation over multiple target points allows for the creation of a linear algebra equation that can be used to find the optimal coil currents. In the form ${\varvec{Ai}} = {\varvec{b}}_{{\varvec{t}}}$ we define a ‘forward field’ matrix ${\varvec{A}}$, a ‘target field’ vector ${\varvec{b}}_{{\varvec{t}}}$ and a coil current vector ${\varvec{i}}$. The forward field matrix contains the magnetic field components $B_{x}$, $B_{y}$ and $B_{z}$ generated by a unit current in each coil evaluated at each of $n = 1$ to $N$ total target field points, and so has dimensions $3N$ rows by $M$ columns. The target field vector ${\varvec{b}}_{{\varvec{t}}} \user2{ }$ has dimensions $3N$ rows by 1 column and the current vector ${\varvec{i}}$ has dimensions $M$ rows by 1 column. The forward field matrix is calculated using the coil parameters, and the target field vector is pre-defined, so we wish to find the current vector which appropriately maps the forward field matrix to the target field vector. To ensure that the coil currents for a given set of parameters are physically manageable, we minimise the norm $||{ }{\varvec{Ai}} - {\varvec{b}}_{{{\varvec{t}}}}||^{2}_{2}$ subject to the constraints that the upper and lower bound ($ub$ and $lb$) of the allowed values of any component $i_{m}$ of the vector ${\varvec{i}}$ is $lb \le i_{m} \le ub$ with the bounds defined by the specifications of the coil driving electronics. The problem is formed as

2 $$\mathop {\min }\limits_{{\varvec{i}}} \,||{\varvec{Ai}} - {\varvec{b}}_{{{\varvec{t}}}}||^{2}_{2} \, {\text{subject }}\,{\text{to}} \,lb\, \le \,i_{m} \, \le ub.$$

To obtain a solution for ${\varvec{i}}$ we use constrained quadratic programming^25^ casting the minimisation in the form

3 $$\mathop {\min }\limits_{{\varvec{i}}} (\frac{1}{2}{\varvec{i}}^{T} ({\varvec{A}}^{T} {\varvec{A}}){\varvec{i}} + \left( { - {\varvec{A}}^{T} {\varvec{b}}_{{\varvec{t}}} } \right)^{T} {\varvec{i}}) \,{\text{subject }}\,{\text{to}}\, lb \le i_{m} \le ub$$

where the superscript ${\varvec{A}}^{T}$ denotes the matrix transpose.

The proximity of the coil to the walls of the MSR means that the magnetic fields produced by the interaction of the coils with the MuMetal walls need to be accounted for when designing a high-performance active-shielding system. These interactions have been investigated previously and can be evaluated using a set of virtual mirror currents produced via reflection of the coil wirepaths in the walls of the MSR^19,26,27^. Recursive reflections of the reflected wirepaths are applied to ensure that the boundary conditions are correctly fulfilled, such that tangential field components are zero on the internal surface formed by the walls of the MSR. To ensure that the magnetic fields produced by the simulated coil designs reflect the real-world case, the effects of interactions up to third order reflections are incorporated into the forward field matrix. We assumed that the coils are displaced from each wall of the MSR by 0.02 m when calculating the position of the reflected elements. The vector sum of the magnetic field produced by each of the reflected elements at each target point forms the magnetic field value ${\varvec{b}}_{{\varvec{m}}} \left( {{\varvec{r}}_{{\varvec{n}}} } \right)$ which is incorporated into the forward field matrix ${\varvec{A}}$.

To optimise coil parameters within this framework we first identified the range of window-coils which could be formed, as each parameter characterising the coils has a maximum value bounded by the dimensions of the MSR. The presence of demagnetisation cabling, cladding panels and flooring reduces the final usable volume onto which we can mount coil panels to $\left( {l_{x} ,l_{y} ,l_{z} } \right) = \left( {2.20 {\text{x}} 2.40 {\text{x}} 2.20} \right) {\text{m}}^{3}$ (coordinate system defined in Fig. 2a,b, y is the vertical direction from floor to ceiling). As we wish for all window coils to share the same parameters, this restricts the permissible values of $L_{c}$ to $L_{c} < \frac{{l_{x} }}{2}.$ For a given value of $L_{c}$ the values of $O$ are then restricted to $O < \frac{{l_{x} }}{2} - \frac{{L_{c} }}{2}.$ We then restrict $H$ for a given value of $L_{c}$ and $O$ such that only the window coils on the $xy$ and $zy$ walls of the MSR have a non-zero $H$, whose values are restricted to $H < \frac{{l_{y} }}{2} - \frac{{L_{c} }}{2} - O.$

For a given set of coil parameters, we find the current vectors which best generate ten different target field vectors: the three uniform field components and (to ensure the magnetic field gradients are well balanced, with symmetry arising from $\nabla \cdot {\varvec{B}} = 0$ and, in the current free region enclosed by the target points, $\nabla \times {\varvec{B}} = 0$) 7 (linear) field gradient components (four longitudinal gradients and three transverse gradients were used, see complete list in Online Appendix 1). For each combination of coil parameters, and each of the 10 field components, $fc$, a value of the quality of the solution $Q_{fc} = ||{\varvec{Ai}} - {\varvec{b}}_{{{\varvec{t}}}}||^{2}_{2}$ is calculated. As optimal coil parameters are likely to be different for different field components, a final combined quality value, $F$, is calculated from the 10 individual values, $F = \mathop \sum \limits_{fc = 1}^{10} \sqrt {Q_{fc}^{2} }$.

The coil parameters which minimise the value of $F$ are found using MATLAB (Mathworks, Natick, MA, USA). The MATLAB constrained minimisation function *fmincon* varies the values of $L_{c}$, $O$ and $H$ according to the constraints above (no negative values of $H$ were considered). The magnetic field from each unit coil was calculated using the Biot-Savart law where the field calculated for a unit current was multiplied by 20 to account for 20 turns of wire. The target field strength for each component was set to 5 nT and 5 nT/m for the magnetic field and magnetic field gradient components respectively. In each case, the target field is calculated over a regular 0.05 m resolution cubic grid ($N = 9261$ target points in total) which spans a volume of 1 × 1 × 1 m^3^ at the centre of the usable portion of the room. The MATLAB optimisation toolbox features a constrained quadratic programming function *quadprog* which was used to solve Eq. (3) and obtain the optimal current vectors to produce each field component for each coil design with currents bound to $- 0.1 {\text{A}} \le i_{m} \le + 0.1 {\text{A}}$. The optimal coil parameters were found to be $L_{c} = 1 {\text{m}}$, $O = 0.55 {\text{m}}$ and $H = 0.08 {\text{m}}$. This design is shown in Fig. 2b.

#### Design iteration

**Figure 3:**
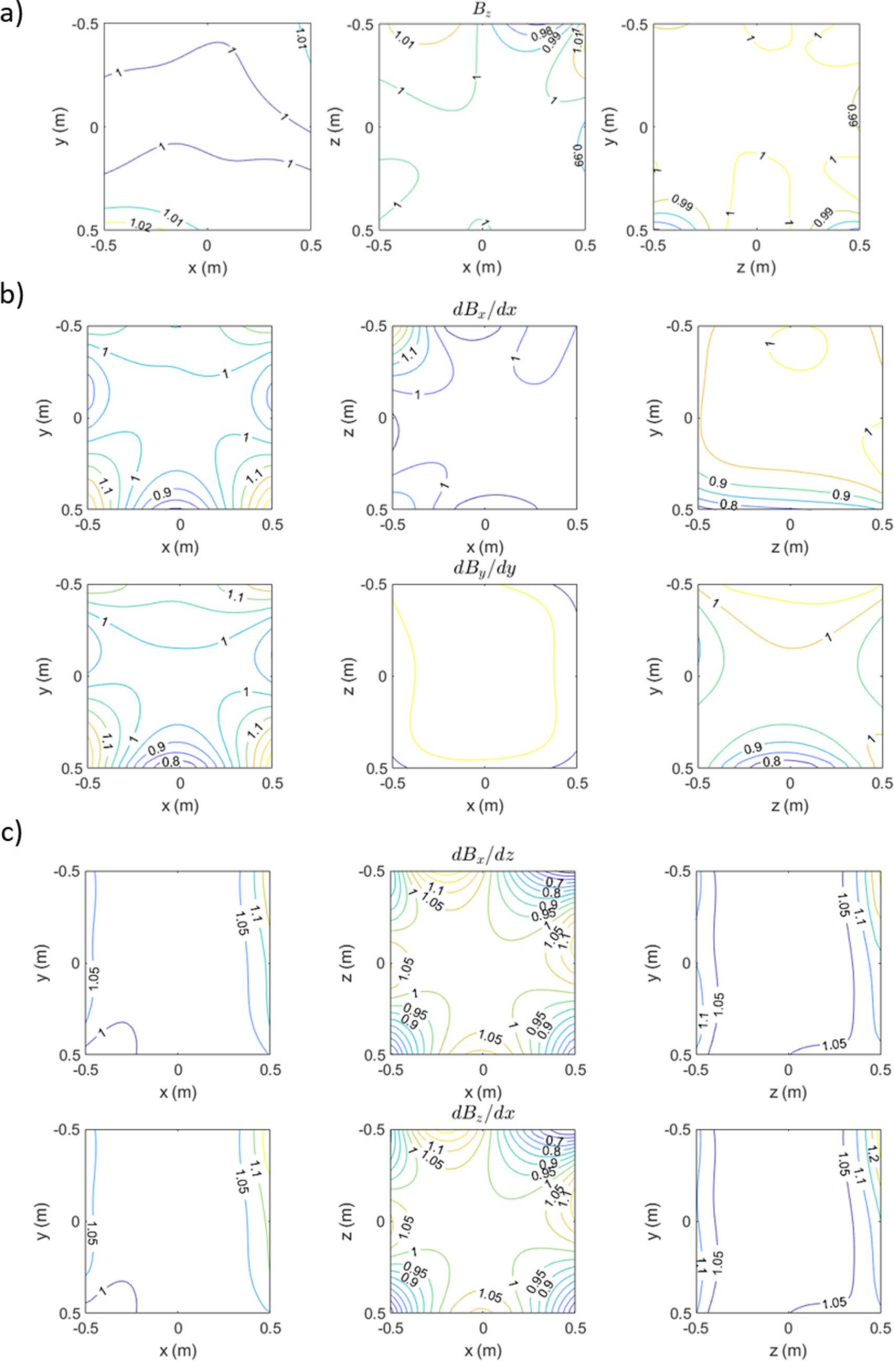
Simulated magnetic field components produced by the window coil active magnetic shielding system. Contours of the magnetic field variation for (**a**) a uniform magnetic field $B_{z}$, (**b**) a longitudinal field gradient $dB_{x} /dx = - dB_{y} /dy$ and (**c**) a transverse field gradient $dB_{x} /dz = dB_{z} /dx$. All contours are shown in three planes (arranged from left to right): z = 0 m |x|, |y|< 0.5 m, y = 0 m |x|, |z|< 0.5 m and x = 0 m |z|, |y|< 0.5 m respectively. The field values at each target point are normalised to the target field or field gradient strength (of 5 nT or 5 nT/m) to show deviation from uniformity. Contours of the remaining magnetic field components are shown in Online Appendix 1.

In practice, the MSR design features several ‘no-go’ areas on the inner walls such as the sites of waveguides for equipment cabling, holes for visual projection and a large access door for participants and experimenters. The location of these features makes the optimised coil design (Fig. 2b) challenging to realise. The optimal parameters were therefore used as a guide to adapt the design to accommodate the no-go regions on the surface of the Light MuRoom structure. Figure 2c shows the adapted coil design. No changes were made on the floor and the right-hand wall. Holes for visual projection were present on the left-hand wall, the rear wall and the ceiling, meaning the coil design on these walls was adapted to feature five coils instead of four. Two rectangular coils were placed either side of the projector hole and a smaller rectangular coil was placed around the projector hole. On the door face, four coils were used with two extended up to the edge of the door whilst two smaller coils were added to the door itself. The 27-coil design was shown to slightly increase (indicating poorer performance) the final quality factor by 2%. Figure 3 shows simulated contours over the target points for an example uniform field ($B_{z}$, Fig. 3a), a longitudinal field gradient ($dB_{x} /dx = - dB_{y} /dy$, Fig. 3b) and a transverse field gradient ($dB_{x} /dz = dB_{z} /dx$, Fig. 3c). The magnetic field at each target point in the field map was normalised to the target field or field gradient strength (5 nT or 5 nT/m) to show deviation from field or gradient uniformity over the target points. We note the high uniformity of the uniform field component, < 3% deviation over the 1 × 1 × 1 m^3^ volume. This degrades to > 20% deviation for the longitudinal gradient (Fig. 3b) and > 30% deviation for the transverse gradient (Fig. 3c). Contours of the remaining field and gradient components are shown in Online Appendix 1.

#### Coil construction

Each coil panel was constructed by laying 0.65 mm diameter insulated copper wire into 3 mm grooves machined into 10-mm-thick plastic panels. The grooves were arranged in a spiral pattern for ease of construction that accommodated 20-turns with the average coil side length across turns corresponding to the optimal coil parameters, as shown in Fig. 2d. Figure 2e-f shows the panel layout. The spiral pattern has minimal effect on the produced field at the centre of the MSR compared to the rectangular wirepaths used in simulation. The coil resistance and inductance vary according to panel size, but the maximum values were 4.99 Ω and 1.25 mH respectively. The panels were mounted onto the walls of the MSR and connected to a junction box which is in turn connected to an electronics cabinet. The electronics cabinet contains nine QuSpin Inc. low-noise voltage drivers (http://quspin.com/low-noise-coil-driver/). Each driver controls three coils and each channel was configured to provide up to ± 68 mA current from an input voltage of ± 10 V. The input voltages to the coil drivers are supplied by a series of National Instruments NI-9264 16-bit DACs which are controlled by a NI-cDAQ-9174 DAQ and LabVIEW.

#### Field nulling

**Figure 4:**
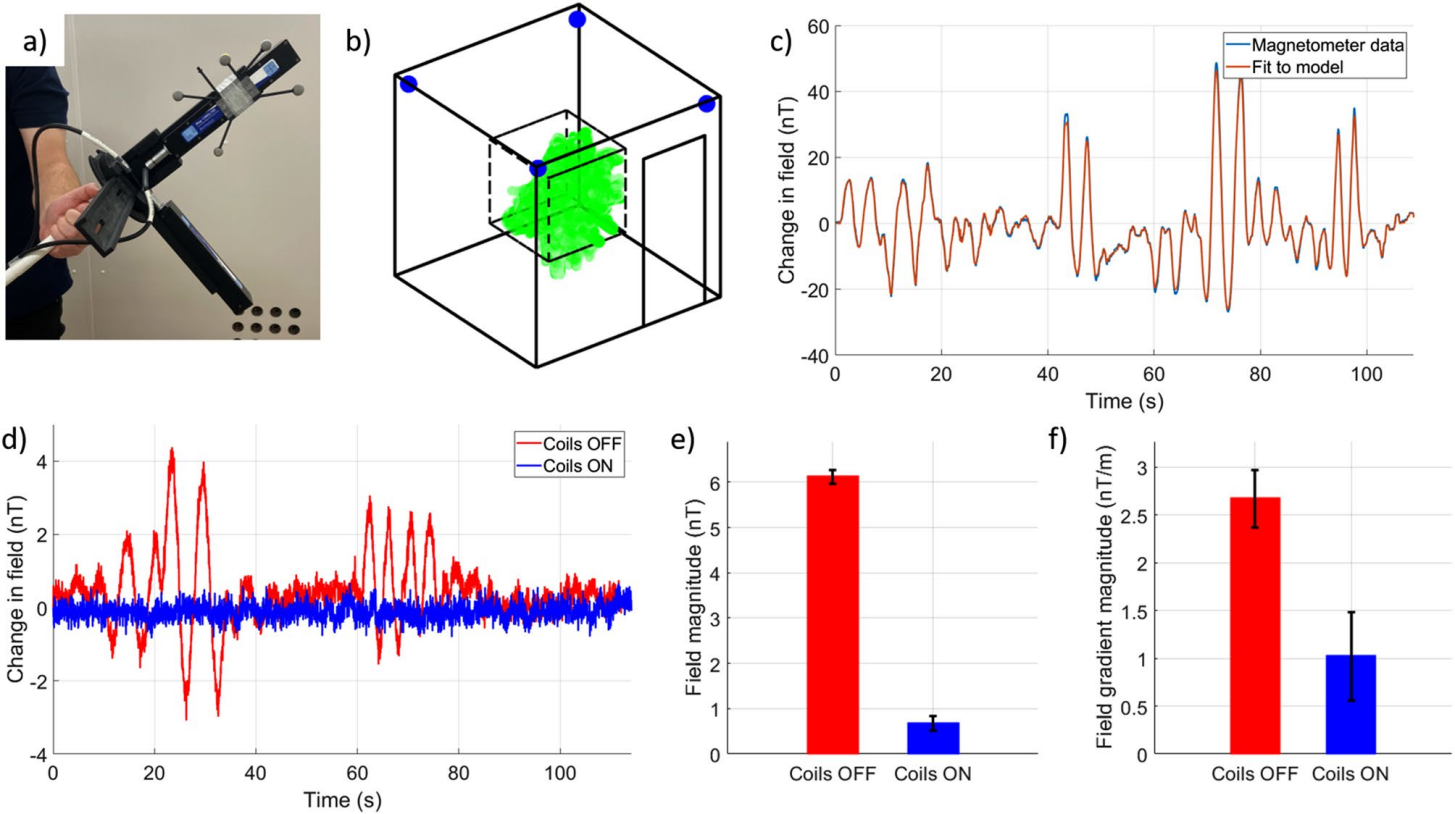
Active compensation of the remnant magnetic field using a field mapping method. (**a**) Two tri-axial fluxgate magnetometers attached to a plastic stick. A series of five infrared reflective markers are also attached to the stick allowing optical tracking of the position and orientation of the sensors within the MSR. (**b**) Schematic of the field mapping setup. The tracking cameras are mounted in the corners of the MSR and highlighted in blue. The dashed black volume shows the central cubic metre volume within which the stick was moved. The green highlighted marks show the path which the fluxgate magnetometers followed during the field mapping process, covering most of the central cubic metre of the MSR. (**c**) Magnetometer data from a single component of one triaxial sensor measured when a single coil was activated. By combining the data from all magnetometers with the optical tracking data a spherical harmonic model can be used to approximate the strength and spatial variation of the field produced by each coil. (**d**) The red trace shows the magnetic field measured by one magnetometer in the MSR with all coils switched off. The magnetic field model of each coil was used to calculate coil voltages which produce the required nulling field. Once voltages had been applied, the mapping was performed again. The blue trace shows the magnetometer data after nulling where similar sensor translations and rotations produce little to no change in the measured field. (**e**) Field mapping and nulling was repeated 8 times. The bar chart shows a consistent remnant field following demagnetisation and a consistent reduction in the RMS magnitude of the three uniform field components found by the model when the compensation is applied. (**f**) A similar reduction is seen in the RMS magnitude of the five field gradient components.

To null the remnant magnetic field, we employed the method described by Rea et al.^28^ which uses optical tracking of a moving array of magnetic field sensors to generate a spherical harmonic model of the magnetic field in the MSR^29^. We mounted two triaxial fluxgate magnetometers (Bartington Mag-13MSL100—low-noise variant with ± 100 µT dynamic range, accuracy < 1 nT, noise < 6 pTrms/√Hz at 1 Hz) along with a set of five infrared reflective markers, onto a plastic stand attached to a plastic stick, as shown in Fig. 4a. Four optical tracking cameras (OptiTrack Flex 13, NaturalPoint Inc., Corvallis, Oregon, USA) were placed in the top corners of the room as shown in Fig. 4b. The cameras track the position of the reflective markers and use the combined coordinates of a series of markers (Fig. 4a) that are fixed with respect to each other (forming a rigid body) to infer six degrees-of-freedom tracking (translation and rotation) of the rigid body with sub-millimetre and sub-1-degree precision.

The fixed positions of the sensitive volumes of the fluxgate sensors with respect to the centre of mass of the rigid body were measured, allowing combination of the optical tracking data with the magnetometer data to produce an accurate fit to a model of the magnetic field. Fluxgate data were collected at 1200 Hz using a NI-9205 16-bit analogue to digital converter (ADC) interfaced with LabVIEW. Optical tracking data were collected at 120 Hz using the OptiTrack Motive software platform interfaced with MATLAB via the Motive NatNet SDK (V3.1). The magnetometer data were low-pass filtered at 10 Hz and down-sampled to 120 Hz to match the sample rate of the optical tracking system. The optical tracking data were also low-pass filtered at 10 Hz. A trigger signal was used to synchronise the two recordings. Both data streams were corrected to reflect the changes in magnetic field and in sensor position and orientation relative to the first timepoint. We chose a third-order spherical harmonic magnetic field model featuring three uniform fields, five field gradients and seven curvature (varying with the square of distance) terms (all terms listed in Table 2), such that the method returns a total of fifteen fit coefficients which describe the relative strength of each spherical harmonic component in the model.

To use the field mapping coefficients to select window coil currents for field nulling, we first mapped the remnant magnetic field (after demagnetisation of the room) over the central cubic metre of the MSR, by performing a series of rotations and translations of the fluxgate magnetometers. This process takes approximately 2 min. The same set of rotations and translations were performed for producing all field maps. An example of the mapped volume is shown in Fig. 4b. We then applied 5 V to a single coil using the DACs and mapped the field again. An example fit to the magnetic field generated by one coil is shown in Fig. 4c. By subtracting the coefficients found for the remnant field from those found when the coil was energised and dividing by 5, we obtain the change in each component of our magnetic field model that is generated by a unit of applied voltage. By repeating for all 27 coils we could construct a coil calibration matrix, which describes the change in each field component generated by a unit voltage applied to each coil. The pseudo-inverse of this matrix can then be used to identify the coil voltages which best produce the field required to cancel the remnant field. The correlation coefficient between our model fit and the measured data was > 0.98 for each coil, suggesting a good model and accurate coil calibration values. The calibration stage takes ~ 1 h to complete but is only performed once.

We investigated the performance of the field mapping and nulling method by first demagnetising the MSR, then mapping the remnant magnetic field and calculating the coil voltages required to cancel this magnetic field. Once these nulling voltages had been applied, we then re-mapped the magnetic field inside the MSR, expecting to see a decrease in the changes in magnetic field experienced by the fluxgates as they move through the same path. The nulling was repeated eight times to assess the repeatability of both the demagnetisation process and the level of field cancellation that was achievable.

## Results

### MSR performance

Table 1 shows the measured shielding factors of the MSR at a range of frequencies within the OPM bandwidth.

**Table 1:** Shielding factors of the MSR at different frequencies measured with a fluxgate magnetometer.

Frequency/Hz	Shielding factor
0 (DC)	10,331 (4.84 nT, calculated relative to a nominal 50 µT vertical field)
0.01	158
0.1	237
1	1230
10	9757
100	8065

### Window coil performance and repeatability

The maximum translation of the centre of mass of the optical tracking markers from the centre of the mapped volume was (x,y,z) (0.32 ± 0.08 m, 0.50 ± 0.06 m, 0.44 ± 0.06 m) and (0.30 ± 0.04 m, 0.48 ± 0.04 m, 0.41 ± 0.06 m) before and after nulling respectively (mean ± standard deviation of the 8 repeats). The maximum rotation of the centre of mass of the optical tracking markers about the origin in the x,y,z axes was (50 ± 10°, 28 ± 3°, 40 ± 10°) and (50 ± 10°, 28 ± 5°, 40 ± 10°). The consistency of movement suggests changes in the magnetic field model are due to a change in the magnetic field of the MSR. Timecourses of field variation measured by one of the magnetometers before and after field nulling are shown in Fig. 4d. A clear reduction in the size of artefacts during sensor movement is shown when the coils are active. Figure 4e shows a decrease in the magnitude of the three uniform components of the spherical harmonic model from $\left| {\varvec{B}} \right| = 6.13 \pm 0.15$ nT to $\left| {\varvec{B}} \right| = 0.67 \pm 0.16$ nT (mean ± standard deviation of the 8 repeats) before and after the coil voltages were applied. Figure 4f shows a decrease in the magnitude of the five field gradient components from $\left| {\varvec{G}} \right| = 2.67 \pm 0.30$ nT/m to $\left| {\varvec{G}} \right| = 1.02 \pm 0.46$ nT/m. Table 2 summarises the change in each of the fifteen components in the spherical harmonic model, reduction is observed in all uniform field and gradient components. Although an increase in some curvature components is observed their strength is ~ 1 nT/m^2^, and the variation with the square of distance means these terms have minimal impact on the field at the noise level of the fluxgate sensors. The average standard deviation ($\sigma$) of the six individual timecourses of fluxgate data used to generate the model fits reduced from $\sigma = 0.84 \pm 0.15$ to $\sigma = 0.24 \pm 0.02$ nT before and after field nulling respectively. The average correlation coefficient ($r$) across the 6 magnetometer channels reduced from $r = 0.96 \pm 0.02$ to $r = 0.45 \pm 0.09$ before and after field nulling. The reduction of the correlation coefficient suggests that the magnetic field is compensated to the noise level of the fluxgates; i.e. little artefact remains in the data which correlates with sensor translations and rotations. These results show that the window coil and field mapping method allow effective compensation of the remnant magnetic field inside the MSR.

**Table 2:** Model fit coefficients before and after nulling for fifteen field components in the spherical harmonic model.

Uniform field components	Field strength (nT) coils OFF	Field strength (nT) coils ON	Ratio
$B_{x} \left( {\hat{\user2{x}}} \right)$	− 0.99 ± 0.18	0.14 ± 0.23	7.1
$B_{y} \left( {\hat{\user2{y}}} \right)$	− 4.09 ± 0.17	− 0.44 ± 0.13	9.3
$B_{z} \left( {\hat{\user2{z}}} \right)$	4.45 ± 0.15	− 0.38 ± 0.24	11.7

Field gradient components	Field strength (nT/m) coils OFF	Field strength (nT/m) coils ON	Ratio
$y\hat{\user2{x}} + x\hat{\user2{y}}$($\frac{{dB_{x} }}{dy} = \frac{{dB_{y} }}{dx}$)	− 1.22 ± 0.24	0.37 ± 0.60	3.3
$z\hat{\user2{x}} + x\hat{\user2{z}}$($\frac{{dB_{x} }}{dz} = \frac{{dB_{z} }}{dx}$)	1.05 ± 0.31	− 0.29 ± 0.44	3.6
$z\hat{\user2{y}} + y\hat{\user2{z}}$($\frac{{dB_{y} }}{dz} = \frac{{dB_{z} }}{dy}$)	2.05 ± 0.40	0.02 ± 0.43	102.5
$- x\hat{\user2{x}} - y\hat{\user2{y}} + 2z\hat{\user2{z}}$($- \frac{{dB_{x} }}{dx} - \frac{{dB_{y} }}{dy} = 2\frac{{dB_{z} }}{dz}$)	− 0.12 ± 0.16	− 0.04 ± 0.16	3.0
$x\hat{\user2{x}} - y\hat{\user2{y}}$($\frac{{dB_{x} }}{dx} = - \frac{{dB_{y} }}{dy}$)	− 0.18 ± 0.32	0.13 ± 0.61	1.4

Curvature components	Field strength (nT/m^2^) coils OFF	Field strength (nT/m^2^) coils ON	Ratio
$6xy\hat{\user2{x}} + 3\left( {x^{2} - y^{2} } \right)\hat{\user2{y}}$	0.49 ± 0.16	0.08 ± 0.45	6.1
$3\left( {x^{2} - y^{2} } \right)\hat{\user2{x}} - 6xy\hat{\user2{y}}$	0.09 ± 0.16	0.37 ± 0.26	0.2
$yz\hat{\user2{x}} + xz\hat{\user2{y}} + xy\hat{\user2{z}}$	0.9 ± 1.9	− 1.4 ± 2.4	0.6
$2xz\hat{\user2{x}} - 2yz\hat{\user2{y}} + \left( {x^{2} - y^{2} } \right)\hat{\user2{z}}$	− 0.47 ± 0.74	− 0.27 ± 0.97	1.7
$- 2xy\hat{\user2{x}} + \left( {4z^{2} - x^{2} - 3y^{2} } \right)\hat{\user2{y}} + 8yz\hat{\user2{z}}$	− 0.15 ± 0.12	− 0.25 ± 0.20	0.6
$\left( {4z^{2} - 3x^{2} - y^{2} } \right)\hat{\user2{x}} - 2xy\hat{\user2{y}} + 8xz\hat{\user2{z}}$	− 0.03 ± 0.15	− 0.11 ± 0.20	0.3
$- 6xz\hat{\user2{x}} - 6yz\hat{\user2{y}} + \left( {6z^{2} - 3x^{2} - 3y^{2} } \right)\hat{\user2{z}}$	− 0.19 ± 0.16	0.02 ± 0.20	9.5

Values quoted are the mean and standard deviation over eight repeat measurements. $\hat{\user2{x}}, \hat{\user2{y}}, \hat{\user2{z}}$ denote the Cartesian unit vectors.

### OPM verification

**Figure 5:**
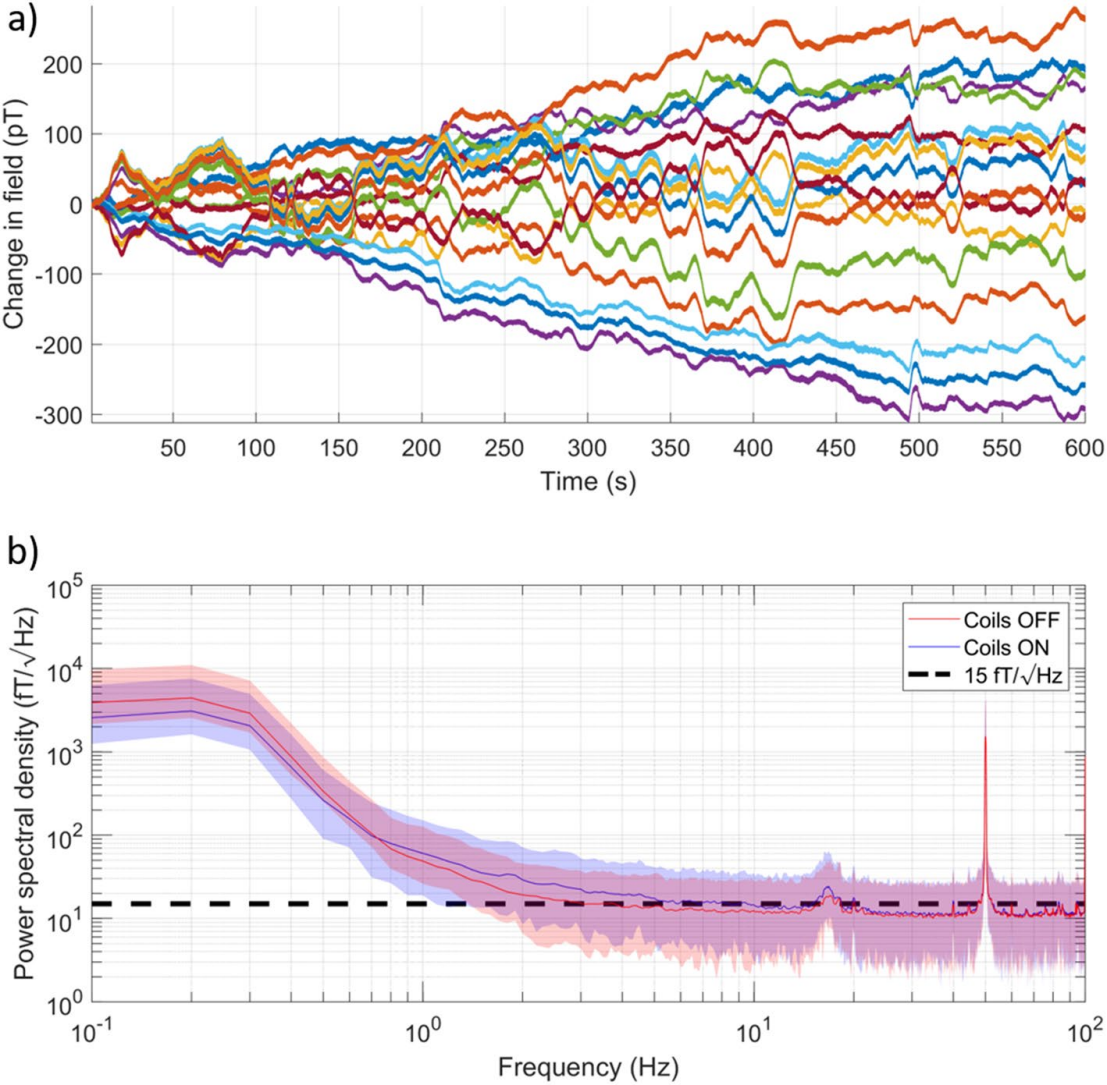
OPM data taken in the Light MuRoom. (**a**) Timecourses of the change in magnetic field experienced by eight dual-axis OPMs (16 total channels) which were placed at the centre of the empty MSR with the window coil system switched on. Each OPM was configured to measure two components of magnetic field. Over 10 min the change in field is ~ 300 pT indicating good shielding performance and a quiet magnetic environment. (**b**) Mean and range (shaded) of the power spectral density of the data collected from the OPMs with and without the window coil system active. The black dashed line indicates 15 fT/√Hz. Data suggests that the MSR is a suitable environment for OPM-MEG, and that the coil system does not add additional magnetic field noise.

Eight QuSpin Zero Field Magnetometers (QZFM, 3rd Generation, Dual-axis variant, sensitivity < 15 fT/√Hz in 3–100 Hz band) were placed at the centre of the room. The MSR was demagnetised prior to the recordings. The QZFMs were each configured to measure two components of magnetic field. Data were recorded for 10 min at 1200 Hz using a NI-9205 ADC. As the coil voltages found in the previous experiments used < 1% of the DACs’ dynamic range, a 2 kΩ resistor was added in series to each coil so that 30% of the dynamic range could be used. This additional resistance also reduces the current noise which is translated into magnetic field noise inside the MSR. Two recordings were taken: with, and without, the window coil system switched on. Figure 5a shows timecourse data recorded from these sensors during the experiment with the window coil switched on. Figure 5b shows the power spectral density of the data for both cases, analysed by using a flat-top window and segmenting data into 10 s chunks, prior to computing the power spectral density using MATLAB’s periodogram function and then averaging the results. The mean PSD is plotted as a solid line (blue/red for the coils on/off) and the range across all channels is noted by the shaded areas. The data show low-frequency field drifts of ~ 300 pT in 10 min. The mean noise level within the frequency bands of interest for neuronal oscillations, (with/without the window coil switched on respectively) were: delta (0.5–4 Hz) 43/38 fT/√Hz, theta (4–8 Hz) 16/13 ft/√Hz, alpha (8–12 Hz) 14/13 fT/√Hz, beta (13–30 Hz) 14/12 fT/√Hz and gamma (30–100 Hz) 12/11 fT/√Hz. The range across sensors is comparable for the two conditions. This performance is likely to provide a suitable environment for OPM-MEG recordings.

## Discussion

Lightweight MSRs, which can be easily sited, are crucial for the widespread deployment of OPM-MEG systems. The design process here involved halving the number of MuMetal layers, reducing the layer spacing and reducing the thickness of the copper compared to an existing OPM-optimised MSR. Each change will have a negative impact on the shielding factor of the MSR but Table 1 demonstrates that despite these changes, high shielding factors are achieved which provide performance that is sufficient to obtain usable OPM data (Fig. 5). We note that the MSR was located at a site with minimal magnetic disturbances, it remains to be seen if this performance would be suitable for a more challenging environment such as a city-centre or hospital setting without additional techniques, such as operating the coils in a constant feedback loop with fixed reference sensors to cancel low-frequency field changes. The rate of change of field that can be generated by driving a coil of inductance $L$ and efficiency $\eta$ with drive voltage $V$ is given by $dB/dt = \eta V/L$. Setting $\eta$ to the maximum field per unit current required by any of the 27 coils to generate a uniform $B_{x}$ -field at the centre of the MSR of 4.6 nT/mA, we find that a rate of field change of 3.5 nT/ms can be achieved with $V = 1$ V. Since $\left| {\frac{{d{\varvec{B}}}}{dt}} \right| \propto \omega \left| {\varvec{B}} \right|$ for a sinusoidal waveform, this allows the generation of a field of more than 5 nT in amplitude even at 100 Hz, which is more than adequate for field cancellation in the frequency range that is relevant for MEG. At higher frequencies (> 1 kHz) the coupling between the magnetic field generated by the coils and the MuMetal shows significant frequency dependence, producing variations in the strength, phase and spatial variation of the fields. Although dynamic operation of the coils would focus on low-frequency effects, operation at higher frequency could be possible via precise modelling of the MuMetal interaction for different frequency regimes or adaptive algorithms could be employed during constant feedback processes. Optimising the copper layer to improve shielding at higher frequencies could also be used to further improve the shielding factor measurements and enable deployment of the MSR in noisier settings.

Electromagnetic coil systems for compensation of the remnant magnetic field of a MSR have formed a key area of development for OPM-based MEG. Previous work has involved the design and construction of bi-planar^14,19^ and Helmholtz-coil systems^8^, each featuring a series of distinct coils that generate known components of magnetic field or field gradient. The compensation of magnetic fields, and low-frequency field drifts, to the sub nT levels required for sensor operation has been achieved over small volumes which span head-mounted arrays of OPMs undergoing limited movements. The incorporation of interactions of produced fields with the MuMetal walls of the MSR has been shown to improve the quality of the field patterns produced by such coils^21^, potentially providing more accurate field nulling. Coil design techniques and open-source packages have been developed for a variety of shield and coil geometries^20,30^. However, the elaborate wirepaths and multiple coil layers required by these systems lead to a complex manufacturing and installation process. The limited spatial extent of region encompassed by previous coil solutions (e.g. two 1.6 × 1.6 m^2^ planes separated by 1.5 m) has had significant impact on the usable region of the MSR, limiting the extent to which a comfortable scanning environment can be achieved.

The window coil system, and field compensation methods we have described have three key advantages over existing techniques: (1) Manufacturing is simplified, with square/rectangular coils that can be flexibly placed on the inner surface of the MSR. (2) Coil calibration and operation is data-driven, accounting for any field imperfections that cannot be accurately modelled. (3) Coils can be configured to produce magnetic fields within a user-defined volume, effectively ‘re-designing’ themselves to adapt to their environment. This flexibility presents unique opportunities. The large compensation volume described here allows for a wide range of participant motion, but coils could also be tuned to compensate smaller volumes such as the OPM-MEG helmet. By continually monitoring head position with optical tracking cameras, and combining field modelling with the data collected by the OPM sensors in the MEG helmet, the coils could feasibly be operated in a constant feedback mode to continually update the location of the shielded volume, a further advantage of a multi-coil system. The OPMs would also be sensitive to the residual field that the fluxgates are unable to detect, thus improving the performance of the field nulling. Dynamic stabilisation in this way would remove the low-frequency ‘drifts’ present in the OPM data shown in Fig. 5a. These advances could enable experiments which required ambulatory motion—a key step towards realising the full potential of OPM-MEG.

## Supplementary Information

Supplementary Information.

## Data Availability

All data generated or analysed during this study are included in this published article and its supplementary information files.
