# Supplementary material for: A lightweight magnetically shielded room with active shielding
Source: Sci Rep. 2022 Aug 9;12:13561. doi: 10.1038/s41598-022-17346-1 (PMC9363499; doi:10.1038/s41598-022-17346-1)
Supplement: Supplementary file 1 — Supplementary Information. [file 41598_2022_17346_MOESM1_ESM.pdf]

## Appendix 1: Contour plots of simulated field components

To inform the optimal coil parameters (Section 2.2) the ability of each coil to produce a series of three uniform field components ($B_x$, $B_y$ and $B_z$), four longitudinal field gradient components ($dB_x/dx = -dB_y/dy$, $dB_x/dx = -dB_z/dz$, $dB_y/dy = -dB_z/dz$, and $dB_z/dz = -0.5dB_x/dx - 0.5dB_y/dy$) and three transverse field gradient components ($dB_x/dz = dB_z/dx$, $dB_y/dz = dB_z/dy$ and $dB_x/dy = dB_y/dx$) was investigated. Figure 3 shows the simulated contours over the grid of target points for three example fields ($B_z$, $dB_x/dx = -dB_y/dy$, and $dB_x/dz = dB_z/dx$). The full set of contours are shown here. The magnetic field at each target point in the field map was normalised to the target field or field gradient strength (5 nT or 5 nT/m) to show deviation from field or gradient uniformity over the target points. We again note the high uniformity of the uniform field components over the 1 x 1 x 1 m$^3$ volume and poorer performance of the field gradient components.

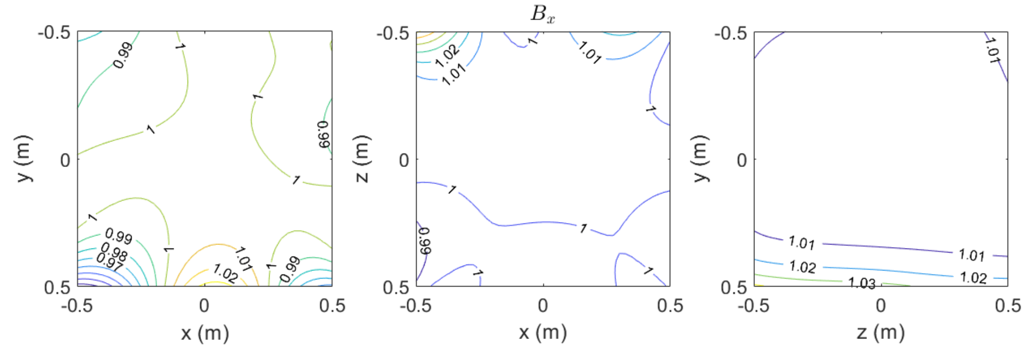


***Figure A1:*** *Contours of the magnetic field variation for the uniform magnetic field component $B_x$. Contours are shown in three planes (arranged from left to right): z = 0 m |x|, |y| < 0.5 m, y = 0 m |x|, |z| < 0.5 m and x = 0 m |z|, |y| < 0.5 m respectively. The field values at each target point are normalised to the target field strength of 5 nT to show deviation from uniformity.*

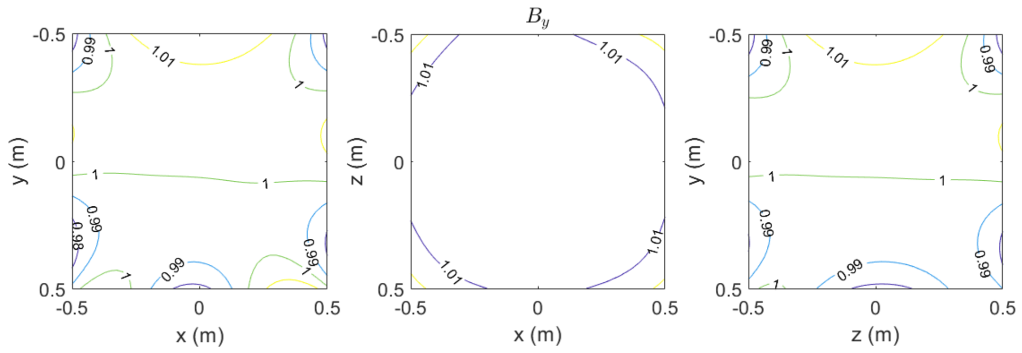


***Figure A2:*** *Contours of the magnetic field variation for the uniform magnetic field component $B_y$. Contours are shown in three planes (arranged from left to right): $z = 0$ m $|x|, |y| < 0.5$ m, $y = 0$ m $|x|, |z| < 0.5$ m and $x = 0$ m $|z|, |y| < 0.5$ m respectively. The field values at each target pointare normalised to the target field strength of 5 nT to show deviation from uniformity.*

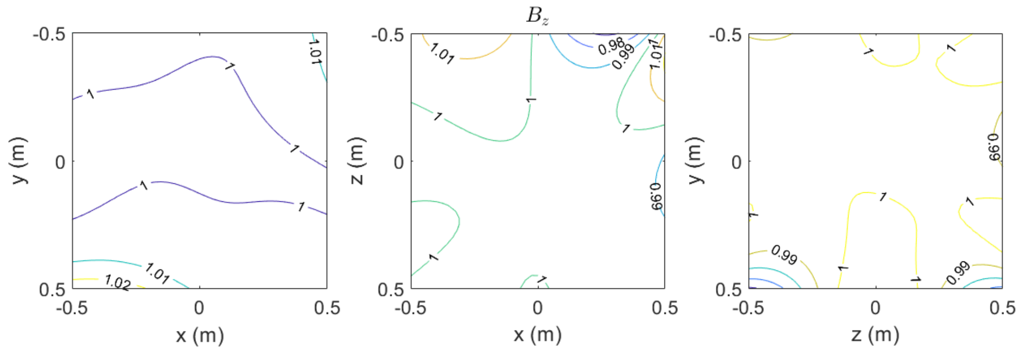


***Figure A3:*** *Contours of the magnetic field variation for the uniform magnetic field component $B_z$. Contours are shown in three planes (arranged from left to right): $z = 0$ m $|x|, |y| < 0.5$ m, $y = 0$ m $|x|, |z| < 0.5$ m and $x = 0$ m $|z|, |y| < 0.5$ m respectively. The field values at each target point are normalised to the target field strength of 5 nT to show deviation from uniformity.*

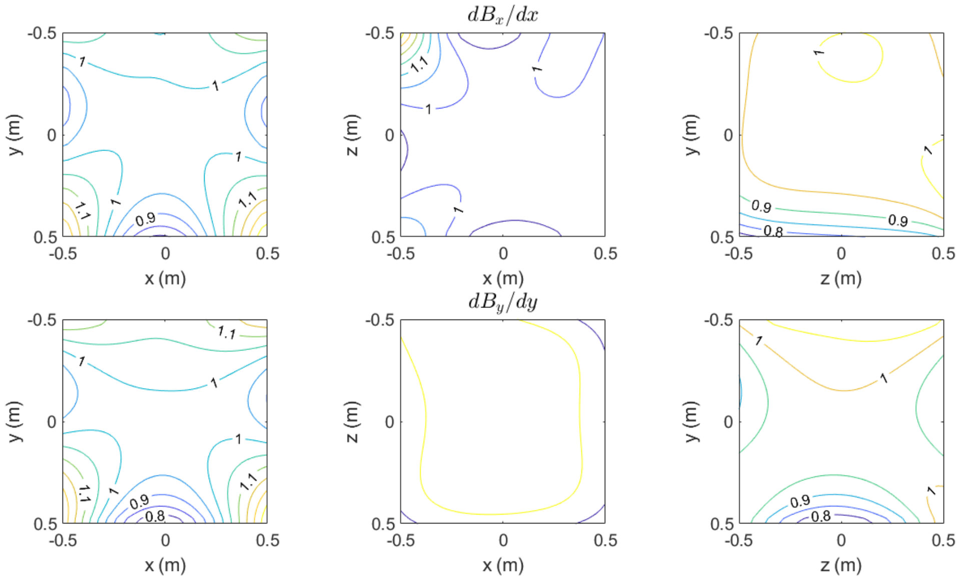


***Figure A4:*** *Contours of the magnetic field variation for the longitudinal magnetic field gradient component $\frac{dB_x}{dx} = -\frac{dB_y}{dy}$. Contours are shown in three planes (arranged from left to right): $z = 0$ m $|x|, |y| < 0.5$ m, $y = 0$ m $|x|, |z| < 0.5$ m and $x = 0$ m $|z|, |y| < 0.5$ m respectively. The field values at each target point are normalised to the target field gradient strength of 5 nT/m to show deviation from uniformity.*

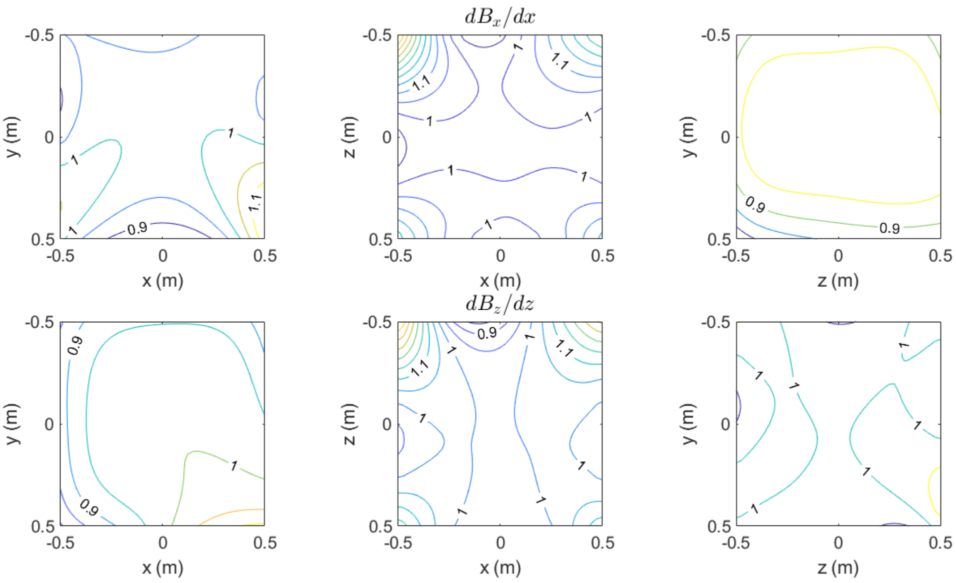


***Figure A5:*** *Contours of the magnetic field variation for the longitudinal magnetic field gradient component* $\frac{dB_x}{dx} = -\frac{dB_z}{dz}$*. Contours are shown in three planes (arranged from left to right):* $z = 0$ *m* $|x|, |y| < 0.5$ *m,* $y = 0$ *m* $|x|, |z| < 0.5$ *m and* $x = 0$ *m* $|z|, |y| < 0.5$ *m respectively. The field values at each target point are normalised to the target field gradient strength of 5 nT/m to show deviation from uniformity.*

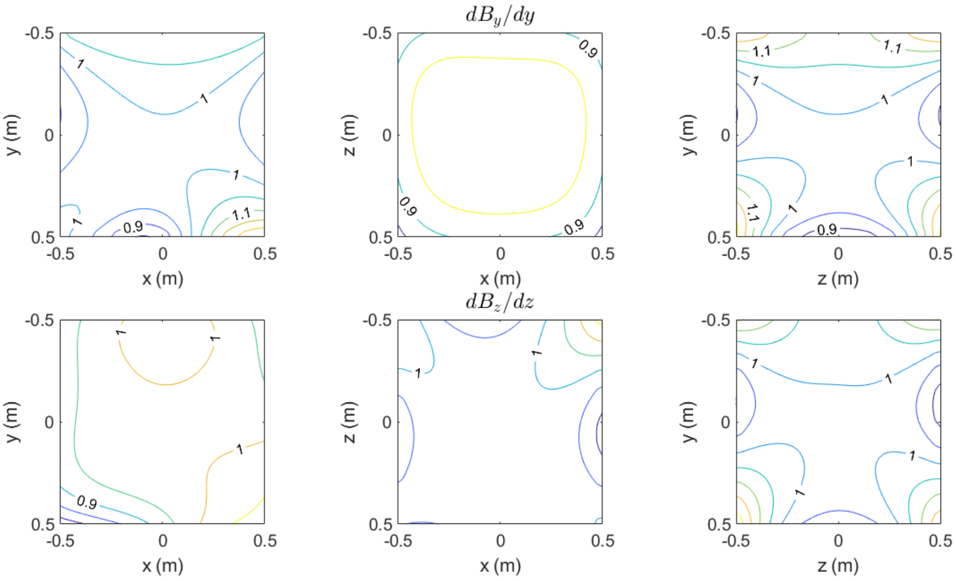


***Figure A6:*** *Contours of the magnetic field variation for the longitudinal magnetic field gradient component* $\frac{dB_y}{dy} = -\frac{dB_z}{dz}$*. Contours are shown in three planes (arranged from left to right):* $z = 0$ *m* $|x|, |y| < 0.5$ *m,* $y = 0$ *m* $|x|, |z| < 0.5$ *m and* $x = 0$ *m* $|z|, |y| < 0.5$ *m respectively. The field values at each target point are normalised to the target field gradient strength of 5 nT/m to show deviation from uniformity.*

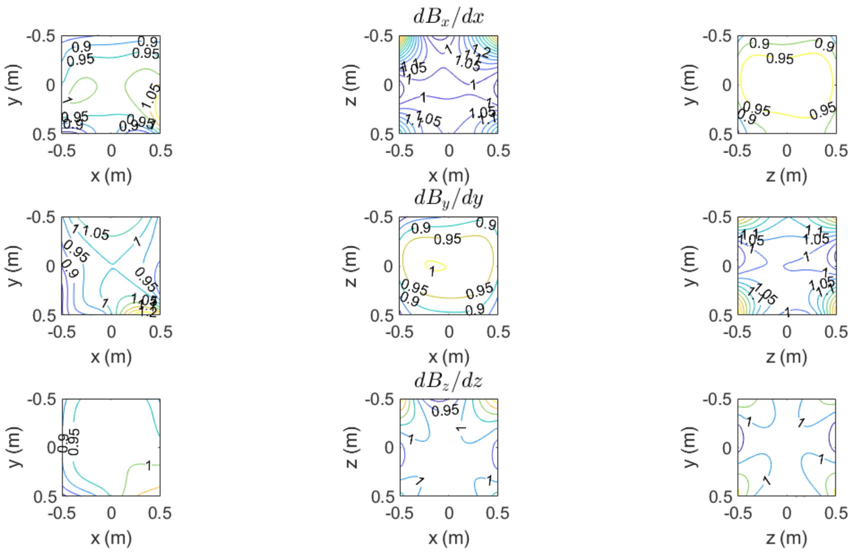


***Figure A7:*** *Contours of the magnetic field variation for the longitudinal magnetic field gradient component* $\frac{dB_z}{dz} = -\frac{dB_x}{dx} - \frac{dB_y}{dy}$. *Contours are shown in three planes (arranged from left to right): z = 0 m |x|, |y| < 0.5 m, y = 0 m |x|, |z| < 0.5 m and x = 0 m |z|, |y| < 0.5 m respectively. The field values at each target point are normalised to the target field gradient strength of 5 nT/m to show deviation from uniformity.*

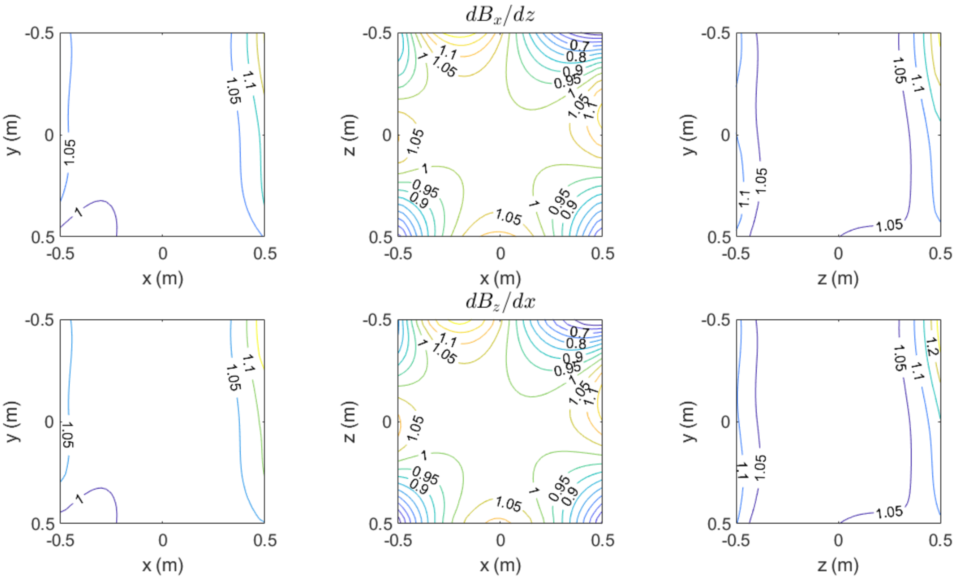


***Figure A8:*** *Contours of the magnetic field variation for the transverse magnetic field gradient component* $\frac{dB_x}{dz} = \frac{dB_z}{dx}$. *Contours are shown in three planes (arranged from left to right): z = 0 m |x|, |y| < 0.5 m, y = 0 m |x|, |z| < 0.5 m and x = 0 m |z|, |y| < 0.5 m respectively. The field values at each target point are normalised to the target field gradient strength of 5 nT/m to show deviation from uniformity.*

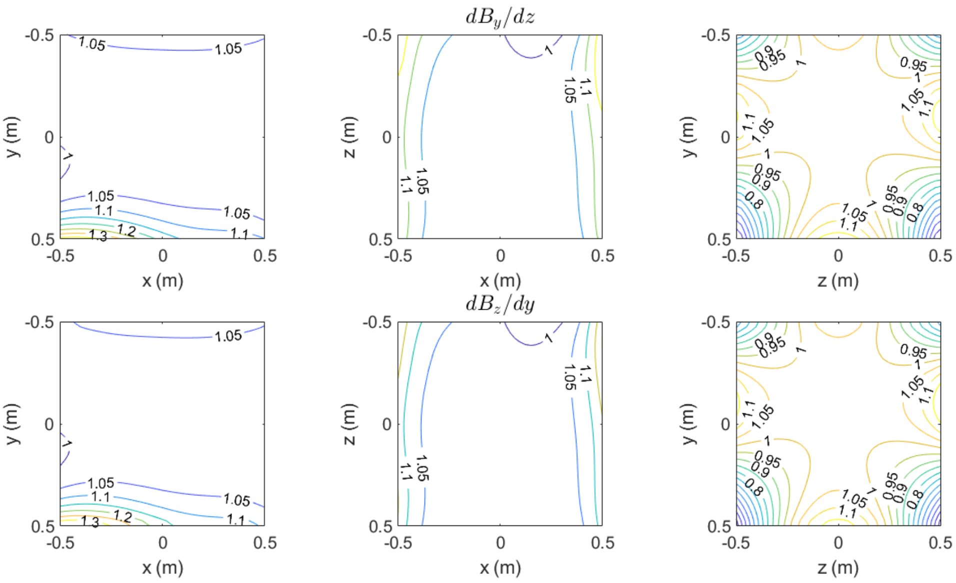


***Figure A9:*** *Contours of the magnetic field variation for the transverse magnetic field gradient component* $\frac{dB_y}{dz} = \frac{dB_z}{dy}$. *Contours are shown in three planes (arranged from left to right): z = 0 m |x|, |y| < 0.5 m, y = 0 m |x|, |z| < 0.5 m and x = 0 m |z|, |y| < 0.5 m respectively. The field values at each target point are normalised to the target field gradient strength of 5 nT/m to show deviation from uniformity.*

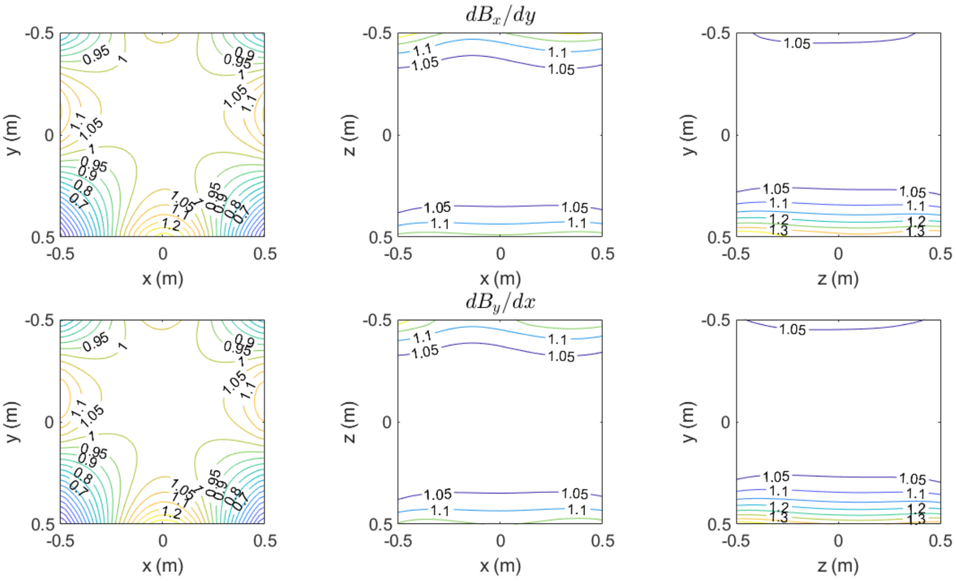


***Figure A10:*** *Contours of the magnetic field variation for the transverse magnetic field gradient component* $\frac{dB_x}{dy} = \frac{dB_y}{dx}$. *Contours are shown in three planes (arranged from left to right): z = 0 m |x|, |y| < 0.5 m, y = 0 m |x|, |z| < 0.5 m and x = 0 m |z|, |y| < 0.5 m respectively. The field values at each target point are normalised to the target field gradient strength of 5 nT/m to show deviation from uniformity.*
